# Composition-driven symptom phrase recognition for Chinese medical consultation corpora

**DOI:** 10.1186/s12911-021-01716-2

**Published:** 2021-12-27

**Authors:** Xuan Gu, Zhengya Sun, Wensheng Zhang

**Affiliations:** 1 University of Chinese Academy of Sciences, Beijing, China; 2 Institute of Automation, Chinese Academy of Sciences, Beijing, China

**Keywords:** Symptom phrase recognition, Named entity recognition, Medical consultation, Composition driven

## Abstract

**Background:**

Symptom phrase recognition is essential to improve the use of unstructured medical consultation corpora for the development of automated question answering systems. A majority of previous works typically require enough manually annotated training data or as complete a symptom dictionary as possible. However, when applied to real scenarios, they will face a dilemma due to the scarcity of the annotated textual resources and the diversity of the spoken language expressions.

**Methods:**

In this paper, we propose a composition-driven method to recognize the symptom phrases from Chinese medical consultation corpora without any annotations. The basic idea is to directly learn models that capture the composition, i.e., the arrangement of the symptom components (semantic units of words). We introduce an automatic annotation strategy for the standard symptom phrases which are collected from multiple data sources. In particular, we combine the position information and the interaction scores between symptom components to characterize the symptom phrases. Equipped with such models, we are allowed to robustly extract symptom phrases that are not seen before.

**Results:**

Without any manual annotations, our method achieves strong positive results on symptom phrase recognition tasks. Experiments also show that our method enjoys great potential with access to plenty of corpora.

**Conclusions:**

Compositionality offers a feasible solution for extracting information from unstructured free text with scarce labels.

## Introduction

The high-speed development of internet is changing the habits of individuals to harvest information and obtain answers to the questions. Nowadays, more and more people try to figure out their physical problems via online consultation with medical professionals^1^. Accordingly, a great number of corpora containing the communications between patients and doctors are accumulated. However, the rare resources of doctors would be hard to satisfy the growing demands for medical services. This prompts the use of medical consultation corpora for the development of medical automatic question answering (QA) systems which have been studied over several decades [1, 2].

The majority of automatic QA systems rely on named entity recognition (NER) as the first step [3, 4]. In clinical domains, named entity recognition refers to the automatic identification of text spans which represent particular entities (e.g., symptoms, diagnoses, medications) [5]. For this study, we focus on the task of symptom phrase recognition, because patient symptoms are integral to health care communications, and diagnostic and therapeutic reasoning. In particular, we concern about the statements which are stored in Chinese medical consultation corpora. Most existing methods for identifying symptoms require either enough manually annotated training data or as complete a symptom dictionary as possible. However, this is often not available in domain specific scenarios. On the one hand, the supervised medical textual resources are quite scarce, because to annotate the data sets needs the experts’ knowledge and experience, which involve high overhead. On the other hand, the spoken language expressions are so diverse that it is difficult to collect all the patients’ descriptions of the symptoms.

**Fig. 1:**
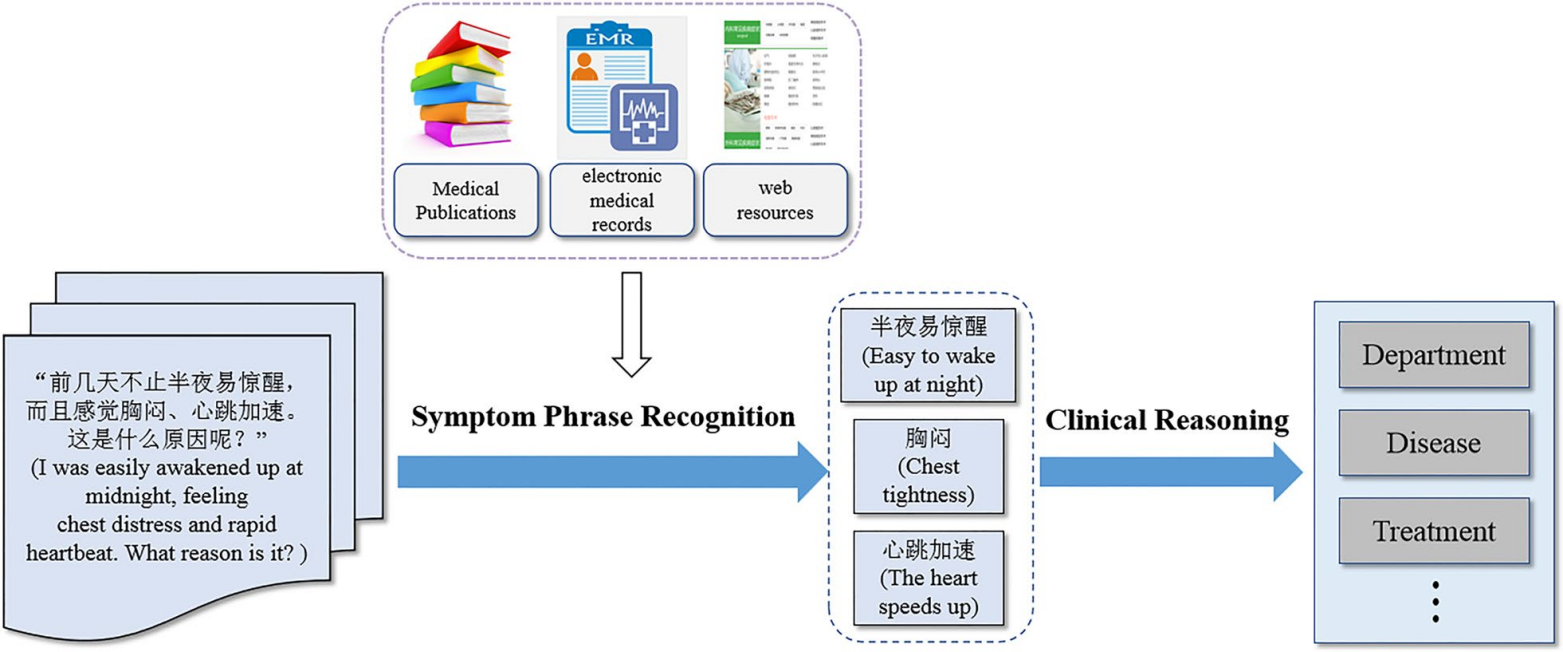
The architecture of the medical QA systems

Like humans, a medical QA system (The architecture of the medical QA systems can be depicted as in Fig. 1.) should be able to leverage known vocabulary to understand the meaning of the processed information. Suppose there is a list of standard symptom phrases used by the domain practitioners, we are interested in recognizing and extracting symptom phrases from patients’ descriptions of what they are experiencing. One straightforward way for exploiting the vocabulary list is to match the entire phrases and decide what symptoms to extract. However, the word sequence repeatedly used by the domain practitioners is an ad-hoc description. Many other wordings in oral expression are used to describe subjective feelings. E.g.  (ENG: “shortness of breath and in a state of discomfort”) describes the typical symptom of  (ENG: “chest distres”). It is often the case that most symptom mentions appearing in the consultation corpus fail to match the symptom phrases in the vocabulary list. An alternative way is to rely on tokenization and match individual words to acquire symptom information. However, this may lead to either incorrect boundaries (e.g., the former three cases in Table 1) or inappropriate collocations (e.g., the latter three cases in Table 1). Note that we use the notation ‘/’ in Chinese sentences to denote the segmentation using the tokenizer.^2^

**Table 1:**
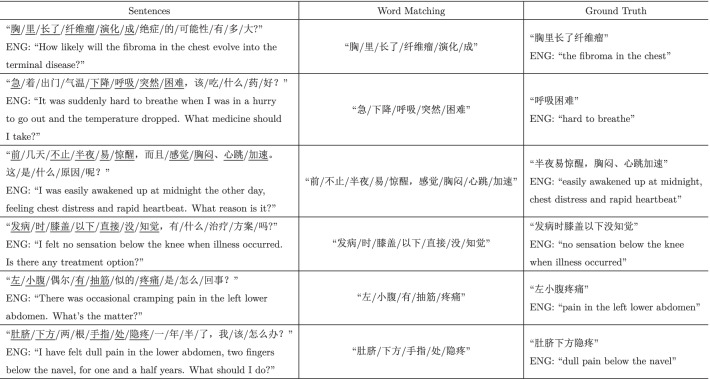
Cases of extraction results by word matching

To tackle these issues, we go beyond word matching and propose a **Com**posi-tion **D**riven (ComD) symptom phrase recognition method for Chinese medical consultation corpora without any annotations. The basic idea is to directly learn models that capture the composition, i.e., the arrangement of the symptom components. We introduce an automatic annotation strategy for the standard symptom phrases which are collected from medical publications, electronic medical records, and web resources. Specifically, we establish a position recognition model based on the relative positions between the symptom components (semantic units of words). Afterwards, we learn the embedding representations for the components, which are then used to estimate the interaction scores between them. By integrating the position outputs and the interaction scores, we are allowed to recognize the symptom phrases in medical consultation corpora. Experimental results demonstrate the feasibility and effectiveness of the proposed method which further improves the overall performance.

The contributions of our paper can be summarized into three aspects:

- We view each symptom phrase in the vocabulary as composition of words and their interactions. This allows us to deduce the symptom phrases that are not seen before, without dependence upon any annotated corpus.
- We incorporate the position outputs and the interaction scores to judge the compositionality between individual words during symptom prediction, which can be viewed as an innovative attempt in the field of data mining.
- Experiments have shown that for symptom phrases recognition tasks, the proposed method can achieve strong positive results, and have great potential with access to increasing online textual corpus.

## Related work

For a medical question answering system, understanding the symptom phrases from the patient’s input is the most critical step in providing an effective solution. A significant way to address the issues of symptom phrases recognition is named entity recognition (NER), which is the task to identify mentions of rigid designators from text belonging to predefined semantic types such as person, location, organization, etc. Over the past few decades, NER has made great strides in a wide range of areas with the help of technologies such as artificial rules, traditional machine learning (ML), and deep learning (DL). Here, we classify these existing NER techniques into three levels from the attributes of methods, and conduct a brief analysis of the pros and cons of some representative strategies related to our work below.

### Rule-based approaches

Some hand-crafted rules, for example, domain-specific gazetteers and syntactic-lexical patterns, are commonly used to design the rule-based NER systems. Kim [6] adopted Brill rule inference method for speech input, making the system generates rules automatically based on Brill’s part of speech markers. In the field of biomedicine, Hanisch et al. [7] proposed ProMiner, which utilizes pre-processed thesaurus to identify protein mentions and potential genes in biomedical texts. Quimbaya et al. [8] presented a dictionary-based NER method based on electronic health records, and experimentally verified that the approach improves recall while having limited impact on precision. UMLS [9] is one biomedical resource which is prepared by medical experts manually. It has a metathesaurus which contains terms and codes from many vocabularies. Luca et al. [10] proposed QuickUMLS: a fast, unsupervised, approximate dictionary matching algorithm for medical concept extraction. Similar approaches identify entities largely through hand-crafted semantic and syntactic rules and then work very well when lexicon can be exhaustive. However, domain-specific rules and incomplete dictionaries make such systems tend to have high precision and low recall, making it impossible to transfer to other domains.

### ML-based approaches

In machine learning, NER is cast into a multi-class classification or clustering task, which learns a model that identifies similar patterns from unseen data. Among the supervised approaches, Bikel et al. [11, 12] proposed an NER system based on Hidden Markov Models (HMM), namely IdentiFinder, to classify names, date, time expressions and numerical quantities. Besides, McCallum et al. [13] proposed a feature-induced method for Conditional Random Fields (CRFs) in NER, which achieves F-score of 84.04% for English by performing on CoNLL03. Krishnan et al. [14] presented a two-stage approach of coupling two CRF classifiers, in which the second CRF utilizes the potential representations from the output of the first CRF. Moreover, there are plenty of supervised NER strategies based on other ML algorithms, Decision Trees [15], Maximum Entropy Models [16] and Support Vector Machines (SVMs) [17] for examples, which have been studied and successfully applied by many scholars. Admittedly, supervised learning algorithms rely on a large amount of annotated data, which is time-consuming and laborious. As a result, unsupervised NER approaches are more desirable. Collins et al. [18] observed that the use of unlabeled data reduces the requirements for supervision to just seven simple seed rules, and then proposed two unsupervised algorithms for the classification of named entities. Nadeau et al. [19] presented an unsupervised gazetteer building and named entity ambiguity resolution system that combines entity extraction with disambiguation based on simple and efficient heuristics. Besides, Zhang et al. [20] proposed an unsupervised method for extracting named entities from biomedical texts by relying on terminologies, corpus statistics and shallow syntactic knowledge, and experiments on two mainstream biomedical databases proved the effectiveness and universality of the method. In the field of semi-supervised approach, Ke et al. [21] proposed using Co-training combining with CRF and SVM on Chinese organization name recognition. Co-training is a semi-supervised learning method, which uses a small amount of tagged corpus and large scales of untagged corpuses for machine learning. Liu et al. [22] proposed to combine a K-Nearest Neighbors (KNN) classifier with a linear Conditional Random Fields(CRF) model under a semi-supervised learning framework to recognize entities for tweets. Actually, the main characteristic of the ML-based approaches is to identify the combination of feature extraction and model selection that work well together for enhanced prediction performance [23–25]. In particular, they extract context features as sources of semantic encoding variability, which may have limitations in the face of less rigid and more flexible spoken language.

### DL-based approaches

In recent years, deep learning, empowered by continuous real-valued vector representations and semantic composition through nonlinear processing, has been employed in NER systems, yielding state-of-the-art performance [26]. The application of neural models for NER was pioneered by [27], where an architecture based on temporal convolutional neural networks over word sequence was proposed. BiLSTM-CRF [28], as the most commonly-used architecture for NER using deep learning, combines BiLSTM and CRF and effectively solves the problem of handling the strong dependence of tags in the sequence ineffectively. Recently, Batbaatar et al. [29] proposed a novel neural network architecture, named semantic-affective neural network (SENN), which utilizes semantic/syntactic and emotional information by using pre-trained word representations. Besides English, there are some studies on Chinese language. Wu et al. [30] studied NER in the Chinese clinical literatures. Zhang et al. [31] proposed an LSTM model of lattice structure for Chinese NER, which encodes the sequence of input characters and all potential words matching the vocabulary. Li et al. [32] pre-trained BERT model on the Chinese clinical domain corpora, and designed a new post-processing way to combine the terminology dictionary with the model and apply radical features to the model on two Clinical Named Entity Recognition (CNER) datasets. Historically, the advantages of deep learning have been less obvious when working with small databases. For example, on the 203,621-word CoNLL-2003 English database, the best DL model, measured by F1 score, outperformed the best shallow model by only 0.4%. In other words, a large amount of annotated data is required to train a good deep learning model.

Although NER has been extensively studied in the biomedical field, symptom phrases seem to have been shelved because there is so little work being done on the subject, especially for Chinese medical texts, which we are mainly discussing here. In our work, we make full use of public resources to mine medical knowledge, and does not require manual annotations. By devising a novel automatic annotation strategy, the proposed approach can save a lot of labour and material costs. More specifically, we combine the position information and the interaction scores between the components of the symptom phrases, and then employ them to identify new symptom phrases in the corpus.

## Proposed method

We begin by introducing a general framework for unsupervised symptom phrase recognition. We then describe three crucial components in detail, i.e., (1) how to characterize the positions of the symptom components, (2) how to calculate the interaction scores between the symptom components, and (3) how to recognize unobserved symptom phrases.

### Overview

For this study, we build a symptom dictionary in advance by aggregating a number of standard symptom phrases from multiple resources (i.e., medical publications, electronic medical records, and web resources). Equipped with the dictionary, we are allowed to mine useful knowledge, which facilitates the downstream task of symptom extraction from medical consultation corpus.

- From the perspective of position, we provide an automatic annotation scheme to indicate demarcation of symptom phrases, followed by characterizing the probabilities of each component (semantic units of words) arranged on different positions within a symptom phrase.
- From the perspective of interaction, we learn the embedding for each component to capture the contextual associations between them. If two components tend to co-occur or appear in similar contexts, they will be mapped into similar word vectors.

**Fig. 2:**
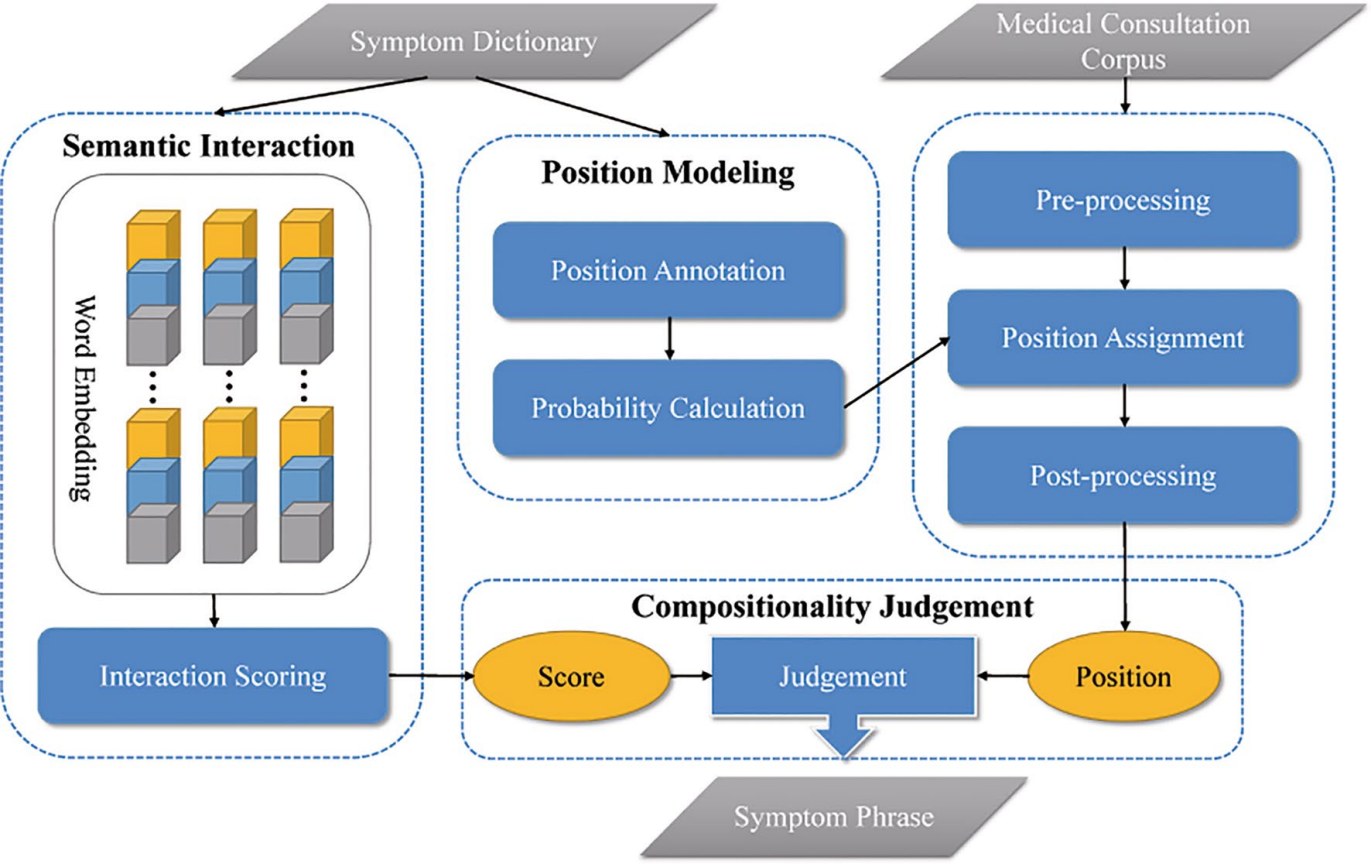
The workflow of ComD

When medical consultation corpus is concerned, we use a basic pipeline to pre-process the texts involved. First, we segmented each text into sentences using a Chinese text analysis tool^3^. We next divided each sentence into words by the tokenizer^4^. After that, we filtered out non-informative words such as extremely common words, rare words, and those that don’t appear in the symptom dictionary. The retained words are then assigned with annotations that contribute to extracting the results. During post-processing, we identify the most probable boundaries of the candidate symptom phrases. Following the principle of compositionality, we integrate the position information and the interaction effect to recognize the symptom phrases that are not seen before. Figure 2 presents the workflow of ComD which comprises three main modules, i.e., position modeling (detailed in Sect. 3.2), semantic interaction (detailed in Sect. 3.3) and compositionality judgement (detailed in Sect. 3.4).

### Position modeling

Different from traditional annotation methods ([33]) which manually annotate each word in the textual corpora, in this task, we consider annotations in the symptom dictionary, which can be realized automatically. As mentioned previously, the symptom dictionary includes a list of standard expressions about patient symptoms. Through observing a variety of Chinese symptom phrases, it was found that there are mainly two types from the view of morphology, i.e., simple words (e.g.  , ENG: “chest distress”) and compound words (e.g.  , ENG: “chest is not smooth”). In addition, a compound word can be split into multiple simple words by the tokenizer mentioned before. Therefore, we adopt the basic annotation signs “BIES” (Begin, Intermediate, End, Single) to represent the position information of a simple word in the phrase. For convenience, we abbreviate a “simple word” as a “word”, unless otherwise stated.

Table 2 shows some basic composition forms of Chinese symptom phrases, and illustrates how the standard symptom phrases are annotated. For example, if there is only one simple word, such as  (ENG: “fever”), then it is annotated as “S”. If there is more than one simple word, such as  (ENG: “persistent low back pain”), then the word  (ENG: “persistent”) is annotated as “B” indicating its beginning position, the word  (ENG: “pain”) is annotated as “E” indicating its end position, and the other word  (ENG: “low back”) is annotated as “I” indicating its intermediate position.

Besides word-based annotations, we can also conduct character-based annotations whose signs “BIES” represent the position information of a character in the symptom phrase, as illustrated in Table 2.

Given a component in a symptom phrase, we count how many times it appears on a particular position. For example, the word  (ENG: “intermittent”) appears 60 times at the beginning and 12 times in the middle. We model the probability of counts as a multinomial distribution $(n, \pi _B, \pi _I, \pi _E, \pi _S)$, where $\pi _B$, $\pi _I$, $\pi _E$, and $\pi _S$ denote the probabilities of four possible positions on each of *n* independent trials. For example, in terms of  (ENG: “intermittent”), there are 72 independent trials, each of which leads to a success for exactly one of the four positions “BIES”. The parameters can be derived based on maximum likelihood estimation. In the above example, the estimations are (0.83, 0.17, 0, 0). Note that these results allow us to infer candidate symptom phrases from the perspective of position arrangements.

**Table 2:**
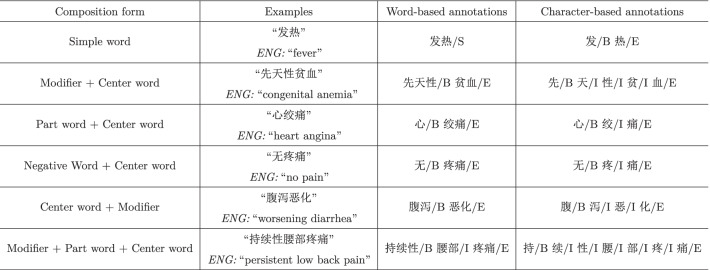
Some basic composition forms of Chinese symptom phrases

### Semantic interaction

We exploit the vector representation of words computed on the symptom dictionary, and implement certain metric to estimate an interaction score between any two words in the embedding space.

**Fig. 3:**
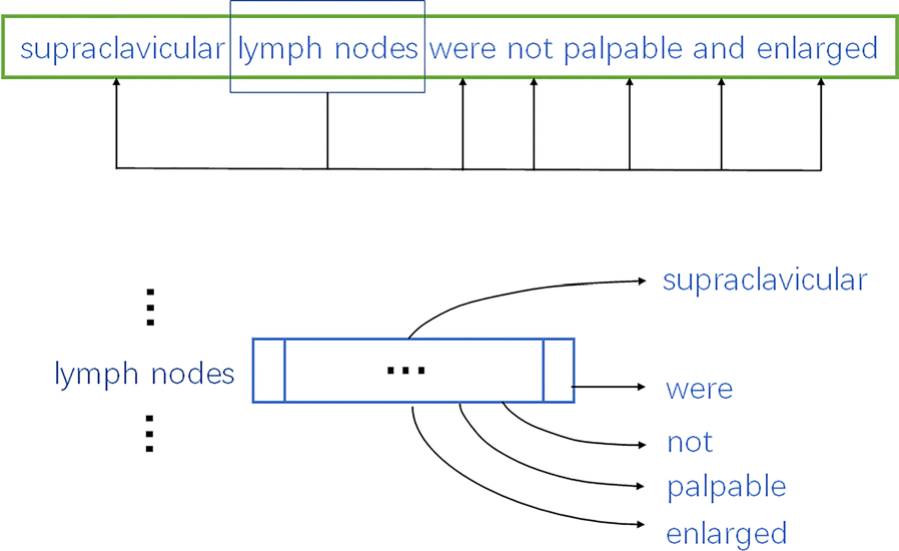
Description of the skip-gram model. The model used in Word2Vec to find an optimal representation to predict the surrounding context of a target word. Consider a standard symptom phrases from the symptom dictionary,  (ENG: “supraclavicular lymph nodes were not palpable and enlarged”). The example highlights the window around  (ENG: “lymph node”), organs that produce immune cells for fighting infections. The target word,  (ENG: “lymph node”), is linked to each of its neighboring words and the pairs are fed into the network. The learning process optimizes the probability of predicting the contextual words of  (ENG: “lymph node”)

(a) *Word embedding* For this study, we build the embedding using the Word2Vec implementation proposed by Mikolov et al. [34], a shallow, two-layer neural network based on a skip-gram model [35]. As is well known, the skip-gram model is directed toward the prediction of the surrounding words given a target word as input (Fig. 3). The learned embedding allows to capture the contextual associations between components in a symptom phrase. In this sense, if two components tend to co-occur or appear in similar contexts, they will be mapped to approximate vectors. Note that there are several neural embedding models which are known to be fully expressive, and which may thus be thought of as more promising candidates for learning word representations [36, 37]. We address whether neural embedding models are able to capture compositions of words and their interactions in the next section.

(b) *Interaction scoring* Once the embeddings are built, we are in a position to predict interaction scores based on the distribution of word vectors in the word embedding. A straightforward way to measure proximity between two components is to use a common distance metric such as Euclidean distance, Manhattan distance or Cosine similarity, and regard them to interact with each other when the metric is within a certain threshold. However, this is problematic due to the fact that the close proximity in high dimensional space does not necessarily imply strong semantic interaction. As high dimensionality often carries rich and diverse semantics, the same distance may arise from different regions of the semantic space. Inspired by [38], we assess the degree of semantic interaction between two components according to the data distribution in their neighbourhood. Intuitively, the less similar neighbours they share, the weaker the semantic interaction is.

Following [38], we first use K-means algorithm [39] to partition the vectors into multiple clusters. Meanwhile, we apply KNN algorithm [40] to find the nearest neighbours. By analyzing the cluster membership of the nearest neighbours, we are allowed to associate each component vector with a discrete probability distribution. In this sense, the discrete probability distribution is derived from the clusters that the neighbours belong to and the corresponding occupancy. Endowed with explicit semantics, the resulting representation helps to support the calculation of interaction scores. We use KL divergence [41] to calculate how much information is lost when approximating one distribution with another. The formula is as follows:

1 $$\begin{aligned} D_{KL}(P_\alpha ||P_\beta )=\sum _{i}P_{\alpha }(i)\log \left[ \frac{P_{\alpha }(i)}{P_{\beta }(i)} \right] , \end{aligned}$$

where $P_{\alpha }$ and $P_{\beta }$ refer to the discrete probability distribution matrices of two components $w_{\alpha }$ and $w_{\beta }$, respectively. For example, for the words  (ENG: “persistent”) and  (ENG: “fever”), $P_\alpha$ represents a discrete probability distribution corresponding to  (ENG: “persistent”) and $P_\beta$ represents a discrete probability distribution corresponding to  (ENG: “fever”).

In order to circumvent the asymmetry of KL divergence, we use a score function based on the JSD, defined as follows [38],

2 $$\begin{aligned} JSD(P_\alpha ||P_\beta )=\frac{1}{2}D_{KL}(P_\alpha ||M)+\frac{1}{2}D_{KL}(P_\beta ||M), \end{aligned}$$

where $$M=\frac{1}{2}(P_\alpha +P_\beta )$$.

In order to measure the interactions between components $w_\alpha$ and $w_\beta$ of interest, we use the scoring function whose range is within [0, 1], defined as follows [38]:

3 $$\begin{aligned} I(w_\alpha , w_\beta )=\exp (-\nu JSD_{\alpha ,\beta }+\gamma ), \end{aligned}$$

Where $\nu$ and $\gamma$ are scaling and offset parameters respectively.

### Compositionality judgement

We combine the position information and the interaction scores to judge the compositionality between individual words. Suppose the words are independent of each other. Let $\pi _B^{w_\alpha }$, $\pi _I^{w_\alpha }$, $\pi _E^{w_\alpha }$, and $\pi _S^{w_\alpha }$ signify the probabilities of “B”, “I”, “E” and “S” positions in term of a given word $w_\alpha$. For an input sentence from patients’ descriptions, we assign each $w_\alpha$ with possible annotations according to $\pi _B^{w_\alpha }$, $\pi _I^{w_\alpha }$, $\pi _E^{w_\alpha }$, and $\pi _S^{w_\alpha }$. Besides “BIES”, we use “O” to represent the “Other” position, indicating that the corresponding component is independent of the extracted results. Actually, the words in a sentence may have more than one annotations. We choose such annotations as candidates, which result in non-overlapping subsequences either assigned with “B” at the beginning and “E” at the end or assigned with “S”. Then, the boundary scoring function ${\varvec{\pi }}(l_1,\dots ,l_q)$ is defined as follows:

4 $$\begin{aligned} {\varvec{\pi }}(l_1,\dots ,l_q)=\sum _{i=1}^q{\varvec{\pi }}^{l_i}, \end{aligned}$$

where ${\varvec{\pi }}^{l_i}$ denotes the score for the subsequence $l_i$. If $l_i$ corresponds to one single component, i.e., $l_i=w_1^i$, then the score is defined as ${\varvec{\pi }}^{l_i}=\pi _S^{w_1^i}$. Otherwise, let $l_i=w_1^i-\cdots -w_k^i$, the score is thus defined as ${\varvec{\pi }}^{l_i}=\pi _B^{w_1^i}\cdot \pi _E^{w_k^i}$.

Note that there may be consecutive occurrences of words with annotation “B”. We treat the leftmost word as the beginning. Similarly, for the consecutive occurrences of words with annotation “E”, we treat the rightmost word as the end. In general, the words that can act as boundaries can also act as intermediate components (e.g.  (ENG: “intermittent”) appears in  (ENG: “intermittent urinary protein”) and  (ENG: “upper limb intermittent dyskinesia”) respectively). This also makes sense from the view of morphology.

After finding the most probable boundaries from the candidates, we are required to determine which intermediate components are useful and indispensable. This is achieved by calculating the interaction scores between each intermediate component and the boundary components. Specifically, if the subsequence is annotated as B-E, its components are both kept and directly combined into a symptom phrase. Otherwise if the subsequence $w_1^j-w_2^j-\cdots -w_k^j$ is annotated as B−I$-$ $\cdots$ $-$E, the component $w_i^j$ assigned with “I” will be discarded if its utility value is less than a certain threshold $\delta$. Formally, the utility function is defined as follows:

5 $$\begin{aligned} {\varvec{\theta }}(w_i^j)=\frac{I(w_1^j,w_i^j)+I(w_i^j,w_k^j)}{2},\quad 1<i<k, \end{aligned}$$

where $I(w_1^j,w_i^j)$ represents the interaction score between the beginning component $w_1^j$ and the intermediate component $w_i^j$, $I(w_i^j,w_k^j)$ represents the interaction score between the intermediate component $w_i^j$ and the end component $w_k^j$, both of which are defined in equation (3).

For example, an input sentence contains a subsequence  (ENG: “An ear infection is so itchy and painful at first”) which is annotated as “B−I−I−I−E”. Let $\delta$ be set to 0.5. Then the intermediate component  (ENG: “at first”) and  (ENG: “so”) are discarded due to their utility values are less than $\delta$. As a result, the extracted symptom phrase is  (ENG: “An ear infection is itchy and painful”). The details are shown in Table 3.

**Table 3:**
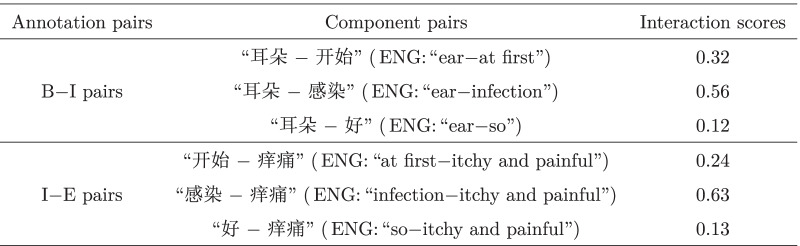
An example of interaction scores between the internals and the boundaries

## Experiments

In this section, we investigate the performance of the proposed method ComD on the medical consultation corpus crawled from public websites. The goals of our experiments are threefold, where one is to investigate the contribution of each module by performing the detailed ablation study, one is to examine whether ComD is able to achieve satisfactory results compared to other baseline approaches and the other is to analyze the effect of the threshold parameter $\delta$.

### Data collection

The datasets consist of the symptom dictionary for training and the medical consultation data for prediction.

*Symptom Dictionary* contains 732,855 symptom phrases which were collected from multiple data sources including medical publications^5^, electronic medical records^6^ and web resources^7^,^8^. They are the conventional/typical expressions used by the domain practitioners.

During pre-processing, we first determined whether the sources have a clear delineation of symptom phrases. If so, we directly loaded them into the dictionary. Otherwise, we predefined several lexico-syntactic patterns to detect symptom phrases in texts. For instance, patterns like “The principal manifestation of NP$_{x}$ is NP$_{y}$”, or “Typical symptoms of NP$_{x}$ include NP$_{y}$” often indicate symptom phrases of the form NP$_{y}$. The punctuation marks in them and duplicate ones are removed subsequently.

*MedConSult* is a collection of nearly one thousand medical consultation records derived from the website^9^. The symptom labels are given by the human annotators. The annotations are used as the ground truth for evaluating the overall performance of the proposed method.

*Curated Data from MedConSult* is a subset of the medical consultation data MedConSult by removing the records that have no annotation signs “I”. In other words, we only kept the records that contain symptom phrases with at least three components. It has been stated that if a record only contains B−E subsequences, we directly combine the boundary components into a symptom phrase without judging their semantic interaction. Therefore, this subset serves the purpose of demonstrating the necessity of each training operation (i.e., position modeling and interaction scoring), and exploring how the recognition performance is sensitive against the variations of the interaction parameter.

### Experimental settings

This section describes the metrics that are used to quantitatively evaluate our method.

*Interaction over Union (IoU)* is the most commonly used metric for comparing the similarity between two strings. The higher the IoU, the closer the extracted result is to the ground truth. In our case, if IoU between the extracted result and the ground truth exceeds a certain threshold $\varepsilon$, the extracted result is assumed to be correct.

*Micro-averaging (Micro)* is an average metric that computes the total number of false positives (FP), false negatives (FN), and true positives (TP) over all consultation records, and then computes the precision, recall, and F$_1$ score using these counts, which are defined as follows,

6 $$\begin{aligned} Micro\text {-}P= & {} \frac{TP}{TP+FP} \times 100 \%, \end{aligned}$$

7 $$\begin{aligned} Micro\text {-}R= & {} \frac{TP}{TP+FN} \times 100 \%, \end{aligned}$$

8 $$\begin{aligned} Micro\text {-}F_1= & {} \frac{2 \times Micro\text {-}P \times Micro\text {-}R}{Micro\text {-}P + Micro\text {-}R} \times 100 \%. \end{aligned}$$

*Macro-averaging (Macro)* is an average metric that treats all records equally, no matter how many symptom phrases they contain. Specifically, it computes the precision and recall independently for each consultation record $s\in S$, and then take the average over the size of the set *S*. The results are then combined to obtain the $F_1$ score.

9 $$\begin{aligned} Macro\text {-}P= & {} \frac{\sum _{s\in S} P_s}{|S|} \times 100 \%, \end{aligned}$$

10 $$\begin{aligned} Macro\text {-}R= & {} \frac{\sum _{s\in S} R_s}{|S|} \times 100 \%, \end{aligned}$$

11 $$\begin{aligned} Macro\text {-}F_1= & {} \frac{2 \times Macro\text {-}P \times Macro\text {-}R}{Macro\text {-}P + Macro\text {-}R} \times 100 \%, \end{aligned}$$

where $P_s$ and $R_s$ denote the precision and recall of the consultation record *s* respectively.

#### Baseline methods

We consider such a setting that only a symptom dictionary including numerous standard symptom phrases is available, while there is no annotated dataset for clinically motivated symptom extraction. In this new setting, we compare our proposed method ComD against the well-developed dictionary-based method and the deep learning method which have achieved the current state-of-the-art on the NER datasets.

- ComD: We leverage the symptom dictionary to learn the arrangements of the symptom components. By incorporating the position outputs and the interaction scores, we are allowed to deduce the symptom phrases that are not seen before.
- BERT-CRF [42]: This method obtains its token representation from the pre-trained BERT model, which is then fed into CRF output layer for token-level classification over the NER label set. As the model requires annotated dataset, for a fair comparison, we use the symptom dictionary to retrieve the relevant consultation records and make annotations accordingly.
- BiLSTM-CRF [43]: This is a character-based CNN-BiLSTM-CRF method for Chinese named entity recognition, which enhances Chinese character representations by character glyphs. The annotated dataset used for BiLSTM-CRF is the same as that used in BiLSTM-CRF mentioned above.
- BDMM [44]: This is a commonly used word segmentation method based on the given dictionary, which combines positive maximal matching and reverse maximal matching algorithm.
- Dictionary-based: This is a dictionary lookup method that relies heavily on exact string matching, where the words, and order of words should be exactly the same as the entry in the symptom dictionary.

We implemented ComD in python. To investigate the principle of compositionality, we simplify ComD to the case in which characters are taken as symptom components. We call this method ComD-Character.

#### Parameter settings

We performed a grid search to tune hyperparameter values for ComD. Each of these parameters is varied from low to high at a fixed interval, and the performance on the medical consultation data is measured. Through trial and error tuning in our implementation, the following choices of hyperparameters are preferred: We set the dimension of word embeddings to 500. We used K-means with 500 clusters, and identified the *k*-nearest neighbours, with $k=2000$. We set the scaling parameter $\nu$, offset parameter $\gamma$ and the utility threshold $\delta$ to 7.5, 0 and 0.2 respectively.

### Ablation study

To investigate the individual contribution of our position modeling and semantic interaction in training, we removed them to offer the methods ComD-NoPos and ComD-NoInt. Without position modeling, the components that interact with each other and appear in a sentence are extracted as a symptom phrase. Without semantic interaction, the components within the boundaries are directly combined into a symptom phrase.

Table 4 reports the macro/micro-averaged results on curated data from MedConSult for different IoU thresholds $\varepsilon$ from 0.6 to 1.0, all shown in percentage. As can be seen, the joint framework shows apparently superior performance in terms of macro/micro-average precision, macro/micro-average recall, and macro/micro-average $F_1$ score. For example, ComD outperforms ComD-NoInt by more than 16% when $\varepsilon = 0.6$, and this improvement is even more pronounced when increasing the parameter $\varepsilon$. This affirms the necessity of semantic interaction, and its ability to retain useful and indispensable components within the boundaries. Compared with ComD-NoPos, it can be observed that ComD increases its performance by an even larger margin. This suggests that position modeling gives more influence to the quality of extractions from unstructured text in spoken form.

**Table 4:** Ablation study results on curated data from MedConSult

$\varepsilon$	Method	Macro/%			Micro/%		
		Precision	Recall	$F_1$	Precision	Recall	$F_1$
0.6	ComD	77.82	78.17	77.99	77.62	78.17	77.89
	ComD-NoInt	61.62	61.97	61.80	61.54	61.97	61.75
	ComD-NoPos	16.20	16.20	16.20	16.20	16.20	16.20
0.7	ComD	75.00	75.35	75.18	74.83	75.35	75.09
	ComD-NoInt	51.06	51.41	51.23	51.05	51.41	51.23
	ComD-NoPos	9.15	9.15	9.15	9.15	9.15	9.15
0.8	ComD	61.62	61.97	61.80	61.54	61.97	61.75
	ComD-NoInt	26.41	26.76	26.58	26.57	26.76	26.67
	ComD-NoPos	5.63	5.63	5.63	5.63	5.63	5.63
1.0	ComD	60.92	61.27	61.09	60.84	61.27	61.05
	ComD-NoInt	15.14	15.49	15.31	15.38	15.49	15.44
	ComD-NoPos	2.11	2.11	2.11	2.11	2.11	2.11

### Overall comparison

We now evaluate the performance of the proposed method and other baselines on MedConSult. The detailed results are summarized in Table 5. As can be seen, our method, ComD, empirically leads to significant gains on recall and F$_1$ metrics, with the precision values slightly lower than that of BERT-CRF. Although BERT-CRF is able to achieve the highest precision values, it is not able to retrieve many symptom phrases shown by the low recall values, only a little better than that of dictionary-based methods. We believe that the good performance of ComD is due to an appropriate design of the model according to the compositionality. As a counterpart, ComD-Character does not perform as well as ComD, albeit its performance superior to the other methods. This is because the semantic units of characters can have multiple meanings in different words, and tend to be confusing indicators for symptom boundaries. When one compares performance of ComD and BiLSTM-CRF, macro-average precision of BiLSTM-CRF at $\varepsilon = 0.7$ appears to be comparative, but the others are relatively poor. BiLSTM-CRF has shown promise in learning Chinese character embeddings. However, it suffers from the training issue: we use symptom dictionary to provide distant supervision so that it could not exploit its full capabilities when facing flexible and variable expressions in the consultation corpus. As expected, BDMM and dictionary lookup methods are inferior to machine learning algorithms. The poor results can be explained by the fact that dictionary based methods are incapable of generalizing beyond the provided word sequences and this limitation is unlikely to be fully compensated by better matching techniques (e.g., BDMM). This also suggests that our method effectively utilizes the composition to detect more symptoms in the consultation corpus which were not present in the symptom dictionary.

**Table 5:** Performance comparison of the proposed and baseline methods on the MedConSult

$\varepsilon$	Method	Macro/%			Micro/%		
		Precision	Recall	$F_1$	Precision	Recall	$F_1$
0.6	ComD	41.02	**37.92**	**39.41**	39.01	**21.84**	**28.00**
	ComD-Character	36.23	33.37	34.74	34.13	13.62	19.47
	BERT-CRF	**47.17**	16.57	24.52	**47.16**	15.32	23.13
	BiLSTM-CRF	36.50	29.58	32.68	34.01	8.43	13.51
	BDMM-based	8.23	12.15	9.81	5.92	8.58	7.00
	Dictionary-based	13.52	7.72	9.83	13.76	7.72	9.89
0.7	ComD	33.53	**30.08**	**31.71**	31.11	**17.42**	**22.33**
	ComD-Character	28.36	25.76	27.00	26.30	10.49	15.00
	BERT-CRF	**37.56**	13.05	19.38	**37.44**	12.03	18.21
	BiLSTM-CRF	32.00	25.80	28.57	26.18	7.51	11.67
	BDMM-based	7.14	10.46	8.49	5.10	7.37	6.02
	Dictionary-based	9.51	5.69	7.12	9.48	5.38	6.86
0.8	ComD	27.45	**24.26**	**25.75**	25.23	**14.12**	**18.11**
	ComD-Character	21.81	19.44	20.55	19.78	7.89	11.28
	BERT-CRF	**27.52**	9.48	14.10	**27.45**	8.82	13.35
	BiLSTM-CRF	19.25	14.94	16.82	14.99	3.61	5.82
	BDMM-based	6.17	8.77	7.25	4.22	6.15	5.00
	Dictionary-based	8.65	5.14	6.44	8.72	4.94	6.31
1.0	ComD	25.05	**22.62**	**23.77**	23.37	**13.08**	**16.78**
	ComD-Character	19.42	17.26	18.28	17.61	7.03	10.04
	BERT-CRF	**26.56**	9.11	13.57	**26.55**	8.53	12.91
	BiLSTM-CRF	15.00	12.52	13.65	12.59	3.03	4.89
	BDMM-based	5.94	8.54	7.01	4.10	5.98	4.86
	Dictionary-based	8.65	5.13	6.44	8.72	4.94	6.31

The best result with bold font for each parameter/model/characteristic

**Fig. 4:**
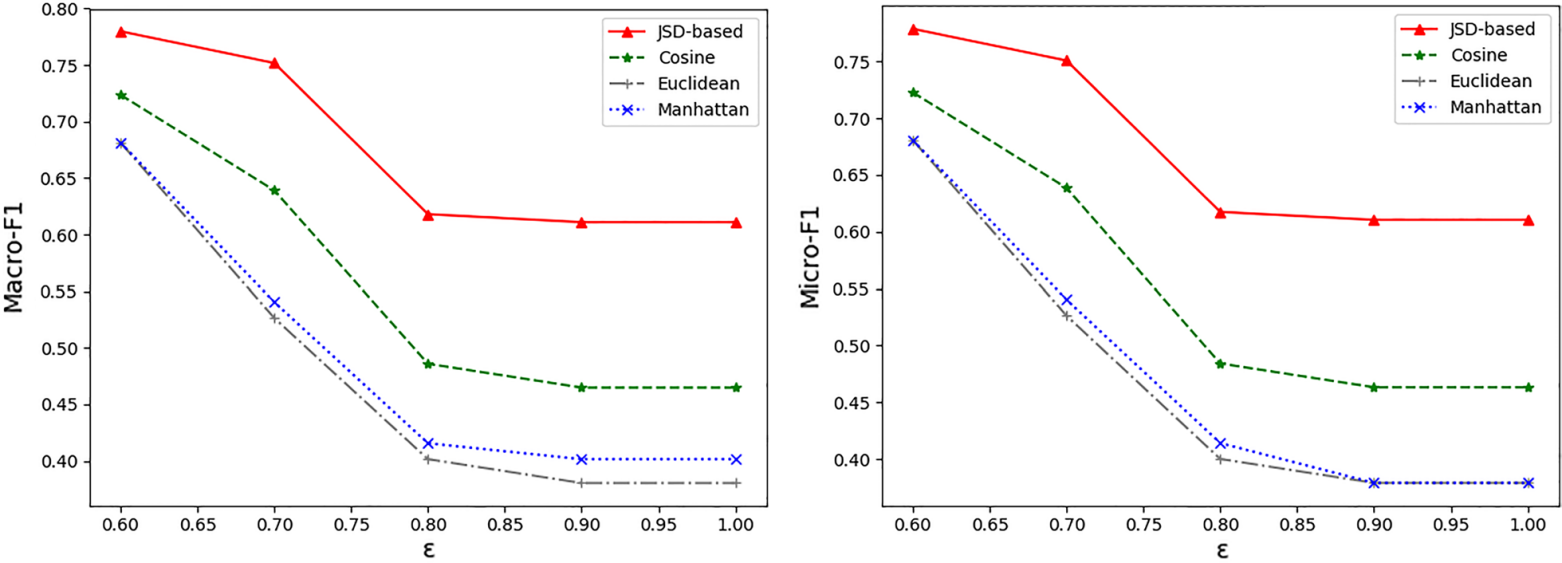
Performance comparison between JSD-based and other distance measures

In addition to the JSD-based scores presented in Sect. 3.3, we also try other distance measures for interaction scoring. Figure 4 plots the curves on macro/micro-average F1 score versus IoU thresholds $\varepsilon$. We find that the results decrease quickly within a certain interval (In this case from 0.6 to 0.85), and then the variations remain slim. This agrees with intuition that a large IoU threshold requires a high match between the extracted symptom phrases and the ground truth. Besides, the larger the value of $\varepsilon$ is, the wider the performance gaps between JSD-based and other distance measures are. This sheds light on the strength of JSD-based metric in capturing the semantic interaction in high dimensional space.

### Sensitivity analysis

**Fig. 5:**
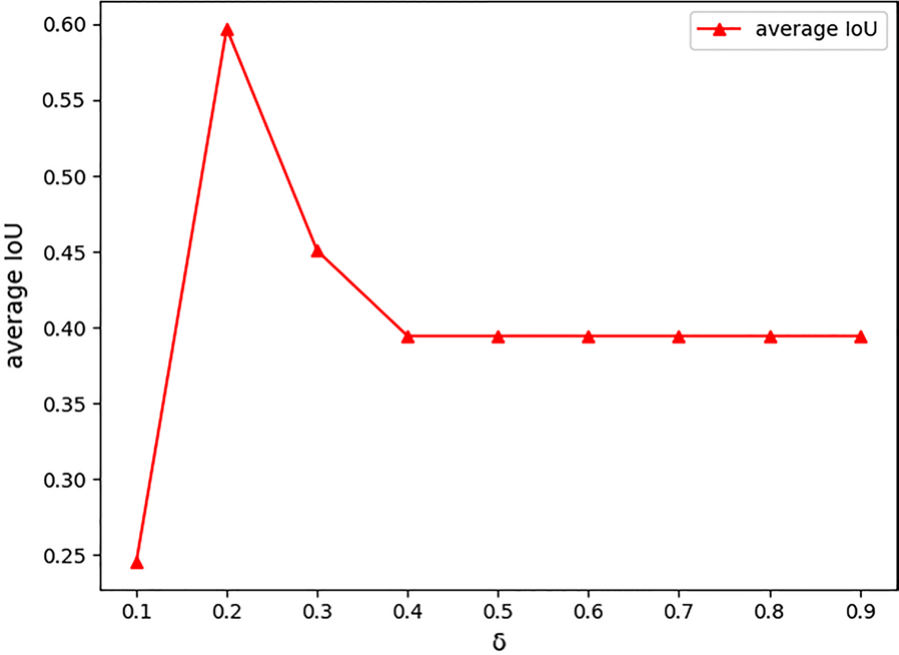
$$\delta$$ sensitivity comparison

We examine how the extraction performance is sensitive against parameter variations. Figure 5 plots the curves on average IoU versus the threshold parameter $\delta$. We observe that the average IoU has a sharp rise when $\delta$ exceeds 0.1, while reaching its peak at 0.2. This can be attributed to the increased ratio of indispensable components needed for symptom recognition. It is noteworthy that when $\delta$ exceeds 0.2, the extraction quality drops quickly, and then seems to remain stable beyond a certain value (in this case around 0.4). This suggests that relatively large $\delta$ would incur performance loss until all the indispensable intermediate components are discarded.

### Case study

In this section, we analyze several representative examples to illustrate the advantages and disadvantages of the proposed ComD, as shown in Table 6. Each case consists of three columns, the first column being the selected cases, the second column being the ground truth, and the third column being the extracted results.

**Table 6:**
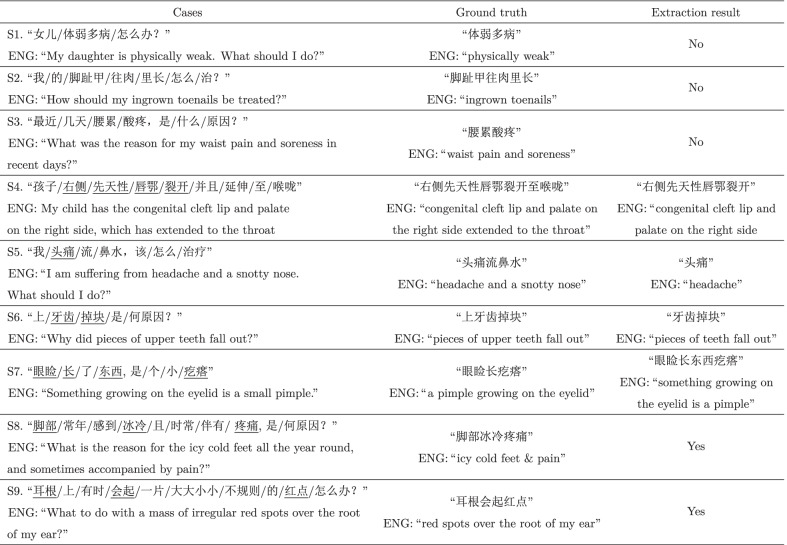
Typical Case

- Sentence S1 makes a claim about a patient experiencing a symptom  (ENG: “physically weak”). In fact, the phrase  (ENG: “physically weak”) is a Chinese idiom which expresses a certain denotation as a whole, and cannot be split into multiple words by the tokenizer. As such, the proposed model fails to recognize the symptom phrase.
- Sentence S2 describes a phenomenon  (ENG: “ingrown toenails”) experienced by a patient, which is further segmented into three words, i.e.,  (ENG: “ingrown toenails”). In our case, they are out-of-vocabulary (OOV) words, and have no word embedding representation learned. Hence, they are ignored when the proposed model is applied.
- Sentence S3 makes a claim about a patient experiencing the symptom  (ENG: “waist pain and soreness”), which composes of two words, i.e.,  (ENG: “waist pain”) and  (ENG: “soreness”). Here,  (ENG: “waist pain”) is an OOV word and ignored during extraction. Although  (ENG: “soreness”) is a valid component of symptom phrases, it rarely appears at the starting position. Without detecting appropriate starting boundaries, the model outputs no extraction results.
- Sentence S4 contains two subsequences relevant to patient symptoms, with the primary symptom being  (ENG: “right side of the congenital cleft lip and palate extends to the throat”). The subsequences are annotated as  and  (ENG: “right side of the congenital cleft lip and palate extends to the throat”). For this case, the proposed model chooses the most probable boundary, i.e.,  (ENG: “right side”) and  (ENG: “cleft”), and leave out the annotated component  (ENG: “to”) and  (ENG: “throat”) beyond the boundaries.
- Sentence S5 makes a claim about a patient experiencing the symptoms  (ENG: “headache and a snotty nose”). However, our method only identifies and extracts  (ENG: “headache) , leaving  (ENG: “a snotty nose”) unrecognized. This is because the two words appearing in the phrase  (ENG: “a snotty nose”) are OOV words, and ignored accordingly.
- Sentence S6 is segmented into five words, i.e.,  (ENG: “Why did small pieces of upper teeth fall out?”), where the former three relate to the symptoms. Through annotating,  (ENG: “upper”) and  (ENG: “teeth”) are both considered as candidate boundaries at the beginning. The proposed model discards the word  (ENG: “upper”) by mistake according to the estimated boundary scores.
- Sentence S7 describes a phenomenon  (ENG: “a pimple growing on the eyelid”) observed by a patient. After finding the most probable boundaries, i.e.,  (ENG: “Something growing on the eyelid is a pimple”), the proposed model calculates the interaction scores between each intermediate component and the boundaries, and then keeps both intermediate components, one of which is yet semantically redundant and unnecessary.
- Sentence S8 and S9 are positive examples that show our model can correctly recognize the symptom phrases. As can be seen, there are disjoint words present in a sentence, which are integral parts of a symptom mention. This is a common phenomenon in colloquial expressions. For example, the words  (ENG: “feet”),  (ENG: “icy cold”) and  (ENG: “pain”) in sentence S8 are disjoint but are all key components of the symptom  (ENG: “icy cold feet & pain”), similar cases in sentence S9. Our model captures such composition and concatenates them as the extraction result.

## Conclusions

In this paper, we explore how using symptom dictionary can facilitate identifying symptom phrases from medical consultation corpus. The basic idea is to learn models for semantic compositionality over linguistic units according to the observed symptom phrases. Our method can not only support computer-assisted diagnosis systems, but can also promote the medical knowledge graph construction. Experimental results prove the superiority of our method. A special emphasis for our future work is placed on more effective design patterns of composing words/characters to form sound symptom expressions. We believe that compositionality provides a feasible solution for extracting information from unstructured free text with scarce labels. Another focus concerns development of computer-assisted medical consultation systems integrated with the proposed symptom recognition method.

## Data Availability

The datasets generated and analysed during the current study are available from the corresponding author upon reasonable request.

## Footnotes

1 These websites include: https://www.haodf.com/, https://www.120ask.com/, and so on.

2 here we use “Jieba” Chinese language segmentation module that is implemented in python https://pypi.org/project/jieba/.

3 https://github.com/blmoistawinde/HarvestText.

4 here we use “Jieba” Chinese language segmentation module that is implemented in python https://pypi.org/project/jieba/.

5 https://item.jd.com/10139850.html, https://item.jd.com/12419020.html.

6 https://www.biendata.xyz/competition/ccks_2021_clinic/data/.

7 https://www.99.com.cn/, http://www.39.net/, https://www.120ask.com/.

8 https://dxy.com/, https://www.guahao.com/, https://www.haodf.com/.

9 https://www.haodf.com/.
